# Surgery

**Published:** 1907-12

**Authors:** James Swain


### SURGERY.

**Excision of the breast for carcinoma** has been attended by better remote results since the adoption of wider removal of skin and lymphatics than was formerly the custom, but there is still much to be accomplished in combating this disease by operation. To those who have not familiarised themselves with modern methods, excision of the breast seems simple enough for anyone to undertake, but the results under these circumstances are deplorable. Several papers have recently been published dealing with this subject, and a consideration of them will show the practice of to-day and the results to be anticipated. At the Massachusetts General Hospital[2] there were 416 cases of primary operation for cancer of the breast during a period of ten years, and of these 376 were traced to a conclusive end-result at an average period of eight years after operation. Sixty-four cases were alive and well, and seven died without recurrence over three years after the operation. Counting in the operative mortality, there were 320 attempts at radical cure, 67 of which, or 20.9 per cent., were successful. Cases in which the tumour was ulcerated, or was adherent to the skin or chest wall, and cases in which the

1 *Deutsches Arch. f. klin. Med.*, 1907, lxxxix. 485.

2 *Surg., Gynec. and Obst.*, 1907, v. 39.

axillary glands were palpably enlarged, gave distinctly less promising results than when these conditions did not exist ; and no case with palpably enlarged cancerous glands above the clavicle, and no case of cancer of both breasts, was cured. It was found that extensive operations with wide removal of skin gave the greatest freedom from local recurrence, but removal of the pectoralis minor appeared to be of slight significance. Recurrence in the scar occurred in less than half the cases. Internal recurrence was most frequent in the lungs, mediastinum, in the axillary and supra-clavicular glands, the liver, and the spine. Nineteen per cent. of those passing the three-year limit without evidence of recurrence, showed recurrence later, and four cases developed recurrence six years or more after the operation. Fifteen cases died as an immediate result of the operation, giving a mortality of 3.6 per cent. on the whole period of ten years ; but the mortality was 5.1 per cent. in the first five years, and 2 per cent. in the last five years. The results which we have just summarised represent the work of twenty different surgeons, and may therefore be regarded as the probable outcome of any large number of cases. Greenough, Simmons and Barney,[1] who have collected these cases in the practice of the Massachusett's Hospital, regard a "complete operation" as one including the removal of the whole breast and axillary contents, the removal of the pectoralis major muscle, and either division or removal of the pectoralis minor. They rightly consider that the amount of skin removed with the breast is a matter of great importance. They contrast the cases in which the wound was directly closed by suture with those in which so large a skin defect was made that a plastic operation was required to close the wound. In 67 out of 160 "complete operations" a plastic operation was performed. Of this number 13 were successful, 19.4 per cent. ; whereas in the 93 complete operations in which the wound was sutured directly there were only 11 successful cases, 11.7 per cent. The difference between these cases was even more marked when local recurrence was considered. Of the 67 cases with plastic operation, no local recurrence in the scar took place in 57.6 per cent., whereas of the complete operations in which the wound was sutured directly, only 44 per cent. remained free from local recurrence.

Having thus considered the results obtained at a general hospital, we may refer to some points in the methods and results of individual operators. Halsted[2] for the past two years has performed the "neck operation" (clearing out the lymphatics above the clavicle) in most cases. He omits it in hopeless cases, in most "duct cancers," and in some carcinomata of adenomatous type in which the axilla at operation is not macroscopically involved. Unless there are special contra-indications, he considers the neck operation should be performed (1) in all cases with

[1] *Loc. cit.* [2] *Ann. Surg.*, 1907, lxvi. 1.

palpable, operable, neck involvement; (2) when the apex of the surgical axilla is involved. When mid-axillary involvement is demonstrable at the operation apical implication is almost certain, and hence (3) in these cases also the neck should be typically cleared of its lymphatics, as high, at the very least, as the bifurcation of the carotid. The influence of early operations and glandular involvements is shown by the fact that he claims a cure of 85 per cent. if the axilla and neck are negative, of 31 per cent. if the axilla is positive and the neck negative, but only of 10 per cent. if both axilla and neck are positive. In considering these statistics Halsted takes the three-year limit as evidence of cure; but Jacobson[1] considers that this limit is too short to determine the end-result. This observer follows Halsted's technique except as to the removal of the supra-clavicular glands, which is only undertaken when there has been apparent invasion of the neck.

Oliver[2] considers that the most potent factor bearing upon prognosis is the character of the growth. The richly cellular, rapidly growing, soft, succulent carcinomata are much less amenable to surgical treatment, even when seen early in the course of the disease, than are the more fibrous, slowly growing, hard varieties of the disease,

Cabot[3] draws attention to the danger of recurrence from the self-inoculation of the wound with cancer cells set free during operation. The possibility of this occurring is a reason for removing breast, muscles and axillary contents in one mass, and for keeping the dissection outside of the lymphatic distribution as far as possible. When a cancer has been cut into for purpose of diagnosis, the opening should be tightly closed before further operation is undertaken, and every precaution should be taken by changing instruments, &c., to avoid inoculation. Where the operation has gone close to the cancer, as through suspicious tissue, Cabot thinks that the application of tincture of iodine to the surface of the wound prevents a quick recurrence when such appears otherwise inevitable. This observer, in common with others, makes it a practice to give each patient a course of X-ray treatment immediately after the operation, with the idea of destroying any bits of cancer that may have escaped removal. For this the exposures to the X-rays are made twice a week for three or four months after operation.

One of the most enthusiastic supporters of clearing the supra-clavicular region as a routine measure is Pilcher,[4] who finds that in ten of the cases in which enlarged supra-clavicular nodes were discovered and removed, three remained free from recurrence. He considers that the point of suspicion is the triangle at the junction of the subclavian and internal jugular veins, where the nodes are found which guard the entrance to the

[1] *Ibid.*, p. 43. [2] *Ibid.*, p. 51. [3] *Ibid.*, p. 57. [4] *Ibid.*, p. 67.

mediastinal lymphatic paths, and to which run not only the lymphatics, which pass up under the clavicle from the axilla, but also an inconstant but not infrequent set of ducts, which run up in the front of the thorax from the mammary region to the base of the neck, down into which they dip after running over the inner end of the clavicle. The deneseness of the deep fascia at the base of the neck, together with the overlying tissue, make it difficult to detect infected nodes by palpation until they have attained some size; but when palpable or visible, the presumption is that the infection is of long standing and of considerable extent, and therefore justifies the gravest prognosis. Pilcher thinks that even then the infection may still be confined to the accessible supra-clavicular group, so that their extirpation may ensure a complete removal of all carcinoma-bearing tissue; but of more importance is the practical recognition of the probability of the presence of infection of the supra-clavicular nodes in every case of breast carcinoma of much duration or extent, and the incorporation into the general plan of operative attack, in all such cases, of an incision into the base of the neck and a systematic removal of all possibly infected tissue, even though there may be no distinct evidence to sight or touch before such incision of the presence of the infection.

From a careful study of fifty cases, Dennis[1] states that after an apparent cure for eighteen years after operation there may yet be a return of the disease in some other organ. In the cases in which no return has been observed, the operation was performed almost without exception within six months of the incipiency of the disease, thus showing the importance of early operation. He, however, points out the fact that in some cases in which the outlook is most unfavourable, as manifested by extensive ulceration, hemorrhage and wide-spread axillary involvement the final results have been entirely satisfactory.

Enough has been said to show that the operation for removal of the breast for carcinoma should not be entered upon without an intimate knowledge of modern principles; and one may call to mind the statement of Ransohoff[2] that "almost everyone who does surgery at all feels himself competent to do a breast operation, which to do properly, in my judgment, is one of the most difficult feats of surgery." From the extracts which we have quoted it can be seen that there is complete unanimity as regards the removal of a wide area of skin, the pectoralis major and the axillary lymphatics. The chief point of difference is as regards the desirability of removing the lymphatics above the clavicle as a routine measure. Some observers state that they always consider this necessary, but it must be remembered that this procedure increases the mortality of the operation. The wiser course perhaps is to be guided by the actual conditions

[1] *Surg., Gynec. and Obst.*, 1907, v. 57. [2] *Ann. Surg.*, 1907, xlvi. 73.

found at the time of operation, and to remove the lymphatics above the clavicle if they are obviously affected, or if the axillary group of glands is affected so high up as to suggest the probability of the infection of the supra-clavicular lymphatics.

As Oliver[1] truly says, the operative treatment of cancer of the breast is far from an ideal method, even with the extensive removals practised at the present time; but the hope for the future lies in better prophylaxis, and in a better knowledge of the nature of the disease.

JAMES SWAIN.

